# Domain-Adaptive Transformer Partial Discharge Recognition Method Combining AlexNet-KAN with DANN

**DOI:** 10.3390/s25061672

**Published:** 2025-03-08

**Authors:** Jianfeng Niu, Yongli Zhu

**Affiliations:** School of Electrical and Electronic Engineering, North China Electric Power University, Baoding 071003, China

**Keywords:** transformer partial discharge, deep learning, Kolmogorov-Arnold networks, domain adaptation, domain adversarial neural networks

## Abstract

The changes in operating conditions of a power transformer can cause a shift in the distribution of partial discharge data, leading to the gradual generation of unlabeled new data, which results in the degradation of the original partial discharge detection model and a decline in its classification performance. To address the aforementioned challenge, a domain-adaptive transformer partial discharge recognition method combining AlexNet-KAN with DANN is proposed. First, the Kolmogorov–Arnold Network (KAN) is introduced to improve the AlexNet model, resulting in the AlexNet-KAN model, which improves the accuracy of transformer partial discharge recognition. Second, the domain adversarial mechanism from domain adaptation theory is applied to the domain of transformer partial discharge recognition, leading to the development of a domain-adaptive transformer partial discharge recognition model that combines AlexNet-KAN with Domain Adversarial Neural Networks (DANNs). Experimental outcomes show that the proposed model effectively adapts transformer partial discharge data from the source domain to the target domain, addressing the issue of distribution shift in transformer partial discharge data with either no labels or very few labels in the new data.

## 1. Introduction

Power transformers are critical components in power systems, and their operating conditions are closely linked to the reliable operation of the entire system. Partial discharge poses significant harm to insulating media, making it a major factor affecting transformer stability and one of the most common transformer faults. Different types of transformer partial discharge vary in their impact on insulation degradation; accurately identifying transformer partial discharge types helps to better understand the characteristics of internal insulation defects in transformers [1].

In the early stages, partial discharge identification in transformers relied primarily on manual judgments by experienced experts, which were time-consuming, labor-intensive, and highly subjective. With the development of machine learning, methods such as Support Vector Machines (SVMs) [2] and Random Forests (RFs) [3] have been applied to partial discharge identification to improve efficiency. However, these methods depend on feature extraction, which can be influenced by subjective judgments, ultimately affecting the model’s recognition accuracy. Deep learning, with its powerful nonlinear fitting and generalization capabilities, enables automatic feature extraction through deep hierarchical structures, establishing direct “end-to-end” mappings from input data [4]. This approach has achieved remarkable success in various recognition domains [5,6]. In partial discharge recognition research, reference [7] used convolutional neural networks (CNNs) to analyze the phase spectrum of pulse sequences for partial discharge type classification, outperforming traditional machine learning methods. Reference [8] employed a blind source separation method relying on a similarity matrix to preprocess the partial discharge ultrasonic time-frequency spectrum, and the processed information was subsequently fed into a convolutional neural network for recognition. AlexNet, an enhanced CNN, reduces gradient vanishing during training and mitigates overfitting, effectively improving recognition efficiency [9]. However, when directly applied to transformer partial discharge identification, AlexNet faces challenges such as reduced flexibility and suboptimal diagnostic accuracy.

The Kolmogorov–Arnold representation theorem asserts that any multivariable continuous mapping can be formulated as a combination of univariate continuous mappings and addition operations. This theorem inspired the development of Kolmogorov–Arnold Networks (KANs) [10,11,12]. KANs replace the linear weights in a Multilayer Perceptron (MLP) with trainable univariate mappings, reducing the number of parameters required while enhancing the function approximation capability [13]. Introducing KANs to improve AlexNet can improve the network’s ability to model complex relationships and enhance classification performance.

Deep learning-based transformer partial discharge recognition models generally assume that the training and testing data are mutually exclusive and come from the same statistical distribution. However, variations in the operating environment of a power transformer lead to distribution shifts in the acquired partial discharge data, causing an increasing diversity of the data and a considerable reduction in the classification accuracy of the original recognition models. Additionally, accurately labeling partial discharge data is challenging, resulting in a large amount of field data with distribution shifts being recorded without labels, which hinders the practical application of deep learning techniques.

Domain adaptation is an effective method for knowledge transfer. It minimizes the distance between data points from different domains based on a defined metric, thereby extracting domain-invariant features from the data [14,15,16,17]. Domain Adversarial Neural Networks (DANNs) bring adversarial learning into domain adaptation, effectively establishing a strong mapping relationship between the source and target domains. This innovation revolutionizes conventional domain adaptation methods and has become a key focus in current domain adaptation research [18]. However, how to better integrate deep learning and DDANs in transformer partial discharge recognition to address the challenges of data distribution shift and the lack of labels for new data remains a key issue, with no relevant research found in the field of transformer partial discharge recognition to date.

In order to tackle the aforementioned challenges, this paper proposes a transformer partial discharge domain adaptation recognition method combining AlexNet-KAN with DANNs. First, KAN is used to improve AlexNet by substituting the fixed activation function in AlexNet with univariate continuous functions, thereby constructing the AlexNet-KAN model. This modification increases the model’s accuracy in identifying partial discharges in transformers. Subsequently, the domain adversarial mechanism is integrated into the AlexNet-KAN model to construct a partial discharge domain adaptation recognition model combining AlexNet-KAN with DANNs, enabling effective adaptation of data from the source domain to the target domain. The results demonstrate that this method achieves high recognition accuracy, effectively addressing the issue of transformer partial discharge data distribution shift and adapting to the continuously increasing diverse unlabeled or sparsely labeled partial discharge data.

## 2. Overall Model Construction

### 2.1. Improvement Based on KAN

**Figure 1:**
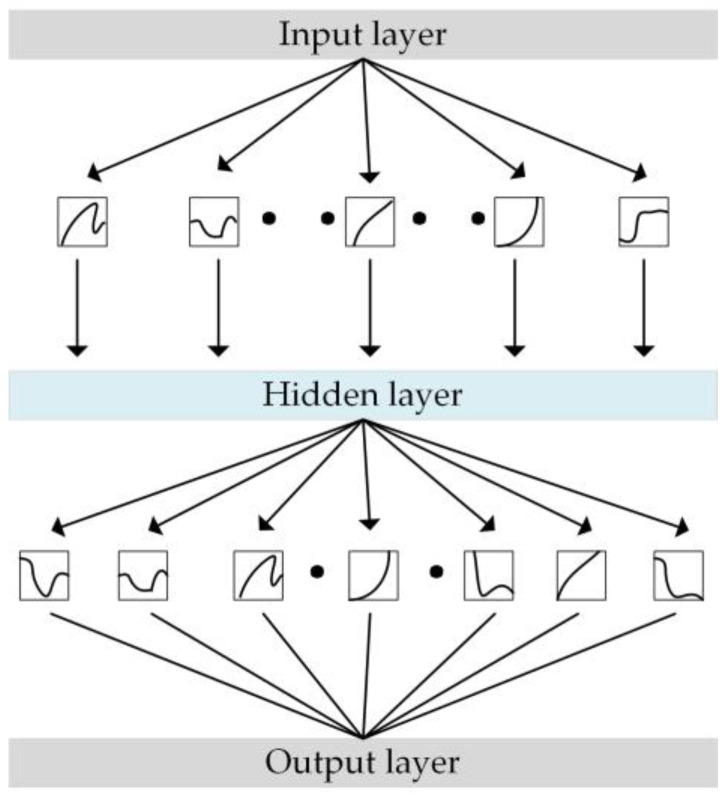
Structure of a KAN.

Compared to MLP, KAN replaces the linear weights in MLP with learnable univariate functions, offering stronger function approximation capabilities and the ability to capture more complex relationships within the data. The structure of a KAN is shown in Figure 1.

This paper improves AlexNet using a KAN by replacing the fixed activation functions in AlexNet with univariate continuous functions, thereby constructing the AlexNet-KAN model. This improvement enhances the expressive capability of the transformer partial discharge recognition model, enabling it to perform better in identifying transformer partial discharges.

### 2.2. Integration with DANN

**Figure 2:**
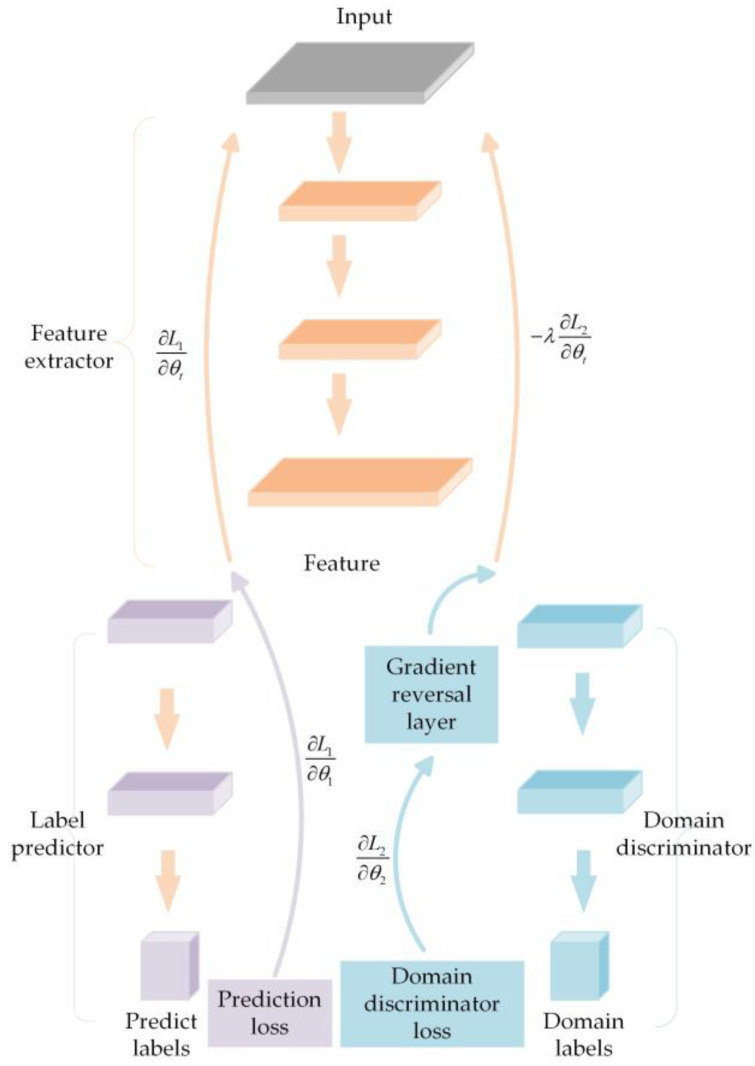
Structure of a DANN.

In domain adaptation, historical data constitutes the source domain, with a large number of labeled samples; new data form the target domain, where samples are either unlabeled or have very few labels. The fundamental framework of domain adaptation includes a feature extractor and a label predictor [19]. To achieve the adversarial effect, DANNs further integrate a domain discriminator into the architecture. The structure of a DANN is illustrated in Figure 2. To optimize the feature extractor while enhancing the domain discriminator’s performance, a gradient reversal layer is incorporated during model training. The gradient reversal layer maintains the input as is during the forward pass, while in the backward pass, it inverts the gradient by scaling it with a negative constant.

The input to a DANN is x, and the output of the label predictor is y. The parameter of the feature extractor $G_{t}$ is $\theta_{t}$, the parameter of the label predictor $G_{1}$ is $\theta_{1}$, and the loss function of the label predictor $G_{1}$ is $L_{1}$; the parameter of the domain discriminator $G_{2}$ is $\theta_{2}$, and the loss function of the domain discriminator $G_{2}$ is $L_{2}$.

The total loss of the domain adversarial network, which is the sum of the label predictor loss and the domain discriminator loss, is expressed as follows:

(1) $$\left\{\begin{array}{l} L(\theta_{1},\theta_{2},\theta_{t})=\sum_{i=1}^{N}{L^{i}}_{1}(\theta_{1},\theta_{t})-\lambda \sum_{i=1}^{N}{L^{i}}_{2}(\theta_{2},\theta_{t})\\ {L^{i}}_{1}(\theta_{1},\theta_{t})=L_{1}(G_{1}(G_{t}(x_{i},\theta_{t}),\theta_{1}),y_{i})\\ {L^{i}}_{2}(\theta_{2},\theta_{t})=L_{2}(G_{2}(G_{t}(x_{i},\theta_{t}),\theta_{2}),y_{i}) \end{array}\right.$$

where λ is the coefficient of the loss function $L_{2}$, and N is the overall count of training samples.

During training, the updates for parameters $\theta_{1}$, $\theta_{2}$, and $\theta_{t}$, based on gradient descent, are as follows:

(2) $$\left\{\begin{array}{l} \theta_{1}\leftarrow \theta_{1}-\alpha \frac{\partial {L^{i}}_{1}}{\partial \theta_{1}}\\ \theta_{2}\leftarrow \theta_{2}-\alpha \frac{\partial {L^{i}}_{2}}{\partial \theta_{2}}\\ \theta_{t}\leftarrow \theta_{t}-\alpha (\frac{\partial {L^{i}}_{1}}{\partial \theta_{t}}-\lambda \frac{\partial {L^{i}}_{2}}{\partial \theta_{t}}) \end{array}\right.$$

where α is the learning rate.

The parameter $\theta_{1}$ of the label predictor reduces the label prediction loss, while the parameter $\theta_{2}$ of the domain discriminator, after the addition of the gradient reversal layer, decreases the domain classification loss. The parameter $\theta_{t}$ of the feature extractor minimizes the label prediction loss while increasing the domain classification loss.

The integration with a DANN enables the model to better align the data distributions of the source and target domains, achieving effective adaptation of data from the source domain to the target domain.

### 2.3. Domain-Adaptive Transformer Partial Discharge Recognition Method Combining AlexNet-KAN with DANN

**Figure 3:**
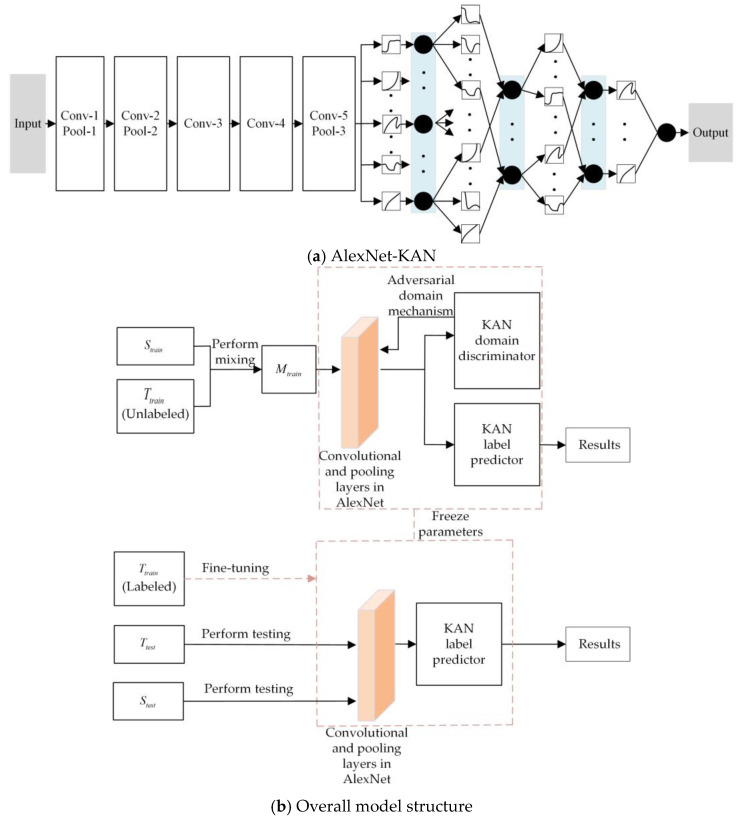
Domain-adaptive transformer partial discharge recognition method combining AlexNet-KAN with DANN.

This paper proposes the use of a KAN to improve AlexNet, enhancing the network’s ability to model complex relationships and boosting the accuracy of transformer partial discharge recognition. Additionally, a DANN is introduced into the field of transformer partial discharge identification, presenting a method that combines AlexNet-KAN with a DANN to address data distribution shifts and the challenge of unlabeled or sparsely labeled new data. The structure of AlexNet-KAN and the overall structure of the method in this paper are shown in Figure 3.

The convolutional and pooling layers of AlexNet serve as the feature extractor in this model, and they are responsible for extracting features from both the source and target domain data. This enhances the KAN label predictor’s ability to make accurate predictions on source domain data while ensuring that the KAN domain discriminator cannot distinguish whether the data originates from the source or target domain. The KAN label predictor is responsible for correctly predicting and outputting labels for source domain data during both training and testing. The KAN domain discriminator, during training, determines whether a sample originates from the source or target domain. When it fails to distinguish domain-specific information for a feature, it indicates that the feature represents domain-independent information.

The overall workflow of the proposed method is as follows:

1. Data Collection: Transformer partial discharge data are collected and phase resolved partial discharge (PRPD) spectra are generated for both the source domain S and the target domain T, which are then compressed into two-dimensional PRPD feature fingerprint images. The datasets are divided into the source domain training set $S_{train}$, source domain test set $S_{test}$, target domain training set $T_{train}$, and target domain test set $T_{test}$;
2. Domain Labeling: The domain labels for the source domain training set $S_{train}$ and the target domain training set $T_{train}$ are one-hot encoded, with the domain label of $S_{train}$ set to 0 and $T_{test}$ set to 1. These two datasets are then merged to form a combined training dataset $M_{train}$;
3. Feature Extraction: $M_{train}$ is input into the proposed transformer partial discharge domain-adaptive recognition model, which combines AlexNet-KAN with DANN, to perform feature extraction. The extracted features are then fed into the model’s label predictor and domain discriminator;
4. Partial Discharge Recognition and Domain Classification: The KAN label predictor performs partial discharge recognition on the transformer data, while the KAN domain discriminator determines whether the input data originates from the source domain or the target domain;
5. Iterative Optimization: The model undergoes iterative optimization until the specified iteration limit is achieved. During this process, the KAN label predictor’s parameters are optimized to minimize partial discharge recognition errors, the KAN domain discriminator’s parameters are adjusted to reduce domain classification errors (facilitated by the gradient reversal layer), and the feature extractor’s parameters are optimized to minimize the KAN label predictor’s loss (ensuring feature discriminability) and maximize the KAN domain discriminator’s loss (ensuring domain-invariant features);
6. Fine-tuning with Labeled Data: If a small amount of labeled transformer partial discharge data are available in the target domain, then these data are used to fine-tune the model to improve the mapping between the source and target domains and further enhance classification accuracy;
7. Testing and Evaluation: The source domain test set $S_{test}$ and the target domain test set $T_{test}$ are input into the model for testing, and the results are analyzed.

## 3. Experiments and Results Analysis

### 3.1. Collection of Experimental Data

**Figure 4:**
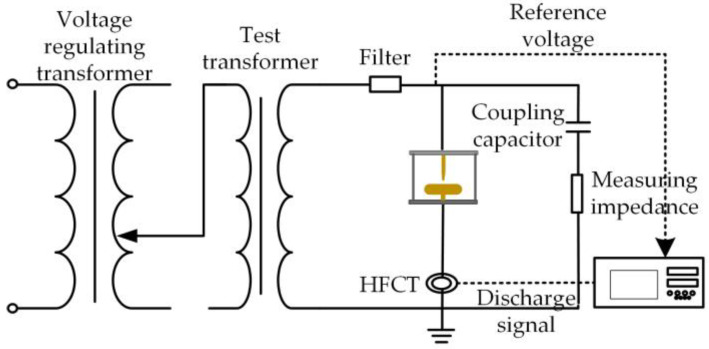
Wiring diagram.

**Figure 5:**
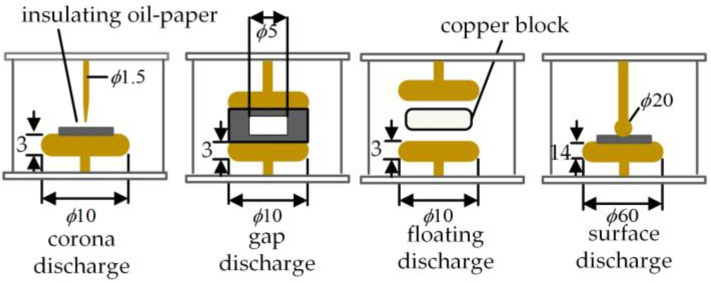
Source domain discharge model.

**Figure 6:**
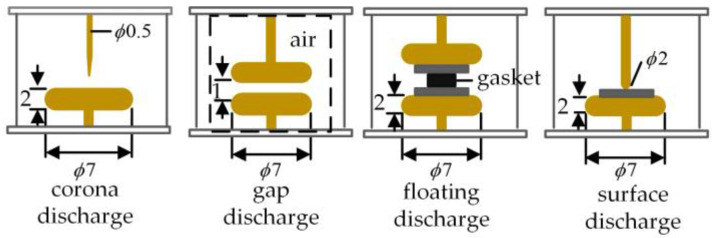
Target domain discharge model.

This manuscript follows the Standard IEC 60270 to establish the partial discharge testing platform for transformers. This study investigates oil-immersed transformers, with the experimental setup illustrated in Figure 4. A source domain discharge model, shown in Figure 5, is first developed, which includes four typical discharge types: corona, gap, floating, and surface discharge. The source domain corona discharge simulates a discharge from a tip near the insulating oil-paper. The gap discharge represents gas bubbles within the oil-paper insulation, the floating discharge uses copper blocks as the components, and the surface discharge is modeled with a spherical-plate electrode (dimensions in millimeters). To simulate the gradual shift in the distribution of transformer partial discharge data and the emergence of more diverse data, a target domain discharge model, shown in Figure 6, is also designed. The target domain corona discharge simulates the discharge from a tip submerged in oil, the gap discharge represents bubble discharges within the oil, the floating discharge uses gaskets as components, and the surface discharge also employs a spherical-plate electrode, but with a different diameter for the spherical electrode compared to the source domain model. Discharge signals are collected using the high-frequency current method from the grounded end of the discharge circuit. Data from the discharge model shown in Figure 5 is used as the source domain data, while the data from the discharge model shown in Figure 6 serves as the target domain data.

**Figure 7:**
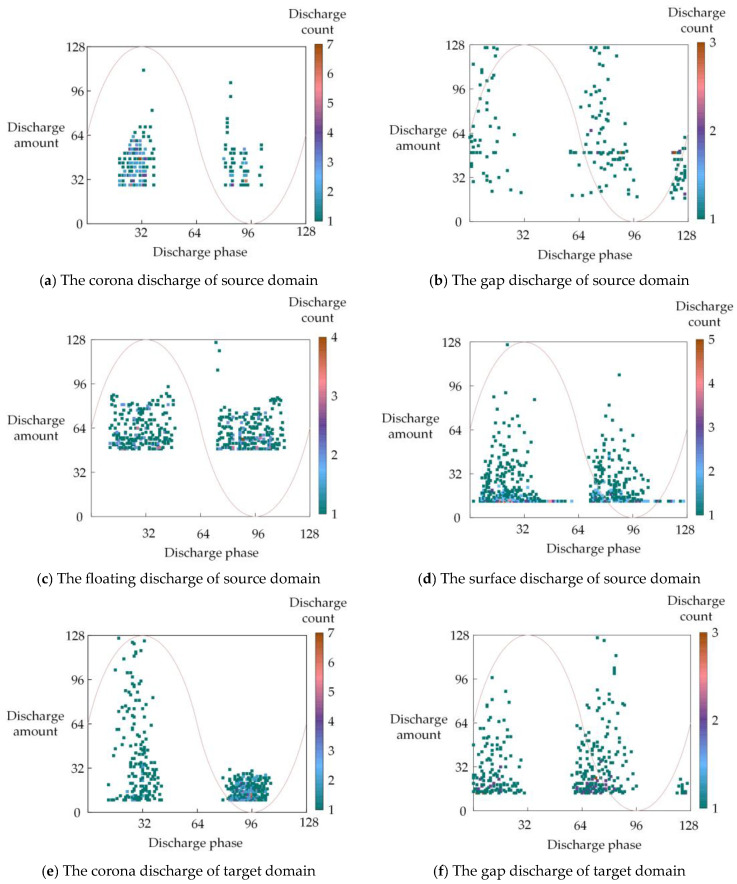

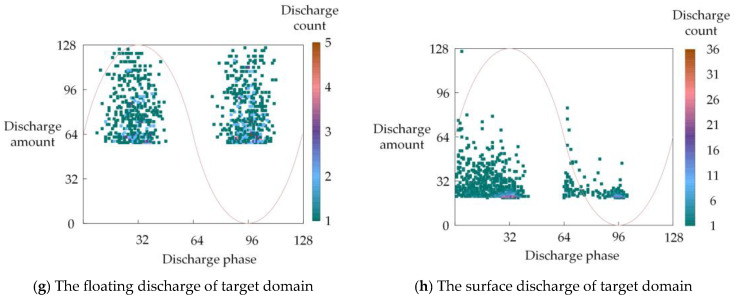
PRPD feature fingerprint diagrams.

The experimental conditions, such as the applied voltage and spacing, were appropriately adjusted, and repeated discharge tests were conducted for both discharge models. The oscilloscope sampling frequency was set to 20 MS/s, and a PRPD diagram was generated every 2 s of partial discharge signal collection. First, the pulse amplitudes of the collected multi-cycle time-domain waveforms were labeled using an adaptive thresholding method, and the moments corresponding to the pulse amplitudes were converted into discharge phases (ranging from 0 to 360°). Next, to alleviate the computational burden on the neural network, the discharge phases (0° to 360°) and discharge amounts (0 to q_max_) in the PRPD spectrum were discretized into 128 equal intervals, resulting in a 128 × 128 two-dimensional matrix. By calculating the values within each region of the feature fingerprint image, the corresponding discharge count was obtained. The PRPD feature fingerprint diagrams for both discharge models are shown in Figure 7.

The PRPD feature fingerprint diagrams of different partial discharge types exhibit distinct differences in discharge phase distribution, discharge frequency, pulse dispersion, and other features. These differences reflect the fundamental characteristics of various discharge mechanisms, which are crucial for partial discharge identification. For the same partial discharge type, however, the PRPD feature fingerprint diagrams from different discharge models reveal both similarities and differences, highlighting the complex influence of factors such as discharge conditions on partial discharge characteristics. In total, the source domain discharge model collects 300 samples for each partial discharge type (200 for training and 100 for testing), resulting in 1200 PRPD feature fingerprint diagrams. The target domain discharge model collects 300 samples for each partial discharge type (150 for training, 100 for fine-tuning, and 50 for testing), resulting in 1200 PRPD feature fingerprint diagrams.

### 3.2. Network Architecture and Parameter Settings

The AlexNet model used in this study consists of eight layers, with parameter settings listed in Table 1. The first five layers of the constructed AlexNet-KAN model follow the same structure as AlexNet, while the fully connected layers are replaced with KAN.

The proposed model is implemented based on the Pytorch framework, with the computing hardware platform being an NVIDIA GeForce RTX 4070 (12 GB), Intel^®^ Core™ i5-13600KF, and 32 GB RAM. The optimization solver used is SGD, and the loss function is the cross-entropy loss function. The parameter λ is set to 0.3 (based on testing, this gave the best performance), with a learning rate of 0.001, batch size of 128, and 300 iterations. During fine-tuning, the training iterations are set to 100, and the other hyperparameters remain the same as during training.

**Figure 8:**
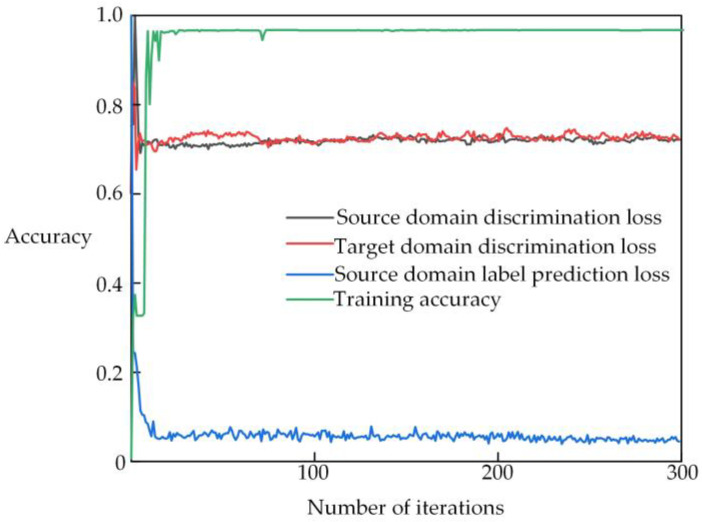
Training process of the model.

The training process of the proposed transformer partial discharge domain adaptation recognition model combining AlexNet-KAN with a DANN is shown in Figure 8. As seen in Figure 8, after a certain number of iterations, the domain discriminator’s loss becomes relatively large and stabilizes, indicating that the features extracted by the feature extractor have achieved domain invariance, enabling the model to better adapt to changes in the transformer partial discharge data distribution as new data is continuously generated. The loss of the model’s label predictor becomes smaller and stabilizes, suggesting that the features extracted by the feature extractor have attained discriminability, allowing the model to correctly identify different types of transformer partial discharge.

To validate the effectiveness of the proposed method in addressing data distribution shift and the issue of missing labels in transformer partial discharge recognition, the following scenarios were set up for comparative experiments:

$Z_{1}$: Training the AlexNet model directly with source domain data;
$Z_{2}$: Training the proposed AlexNet-KAN model directly with source domain data;
$Z_{3}$: Training the proposed model by combining AlexNet-KAN with a DANN with mixed source and target domain data without fine-tuning;
$Z_{4}$: Training the proposed model by combining AlexNet-KAN with a DANN with mixed source and target domain data and fine-tuning the model.

The recognition rates for the source domain and target domain data are shown in Table 2.

As shown in the table above, the recognition accuracy of the AlexNet model on the source domain dataset is 95.32%. However, due to the variations in the distribution between the source and target domain data, the recognition accuracy on the target domain dataset is only 42.46%. The target domain recognition accuracy of the proposed transformer partial discharge domain adaptation recognition method combining AlexNet-KAN with a DANN (after fine-tuning) is 86.85%, the highest among the four scenarios. By comparing $Z_{1}$ and $Z_{2}$, it is evident that after improving the model with a KAN, the accuracy on both the source domain and target domain datasets has increased. This indicates that the improvement with a KAN enhances the model’s ability to model complex relationships, thereby increasing its accuracy in correctly identifying the types of partial discharges. Based on the comparisons between $Z_{2}$, $Z_{3}$, and $Z_{4}$, it can be concluded that by integrating a DANN into the model, the model effectively adapts the data from the source domain to the target domain. The features extracted by the model are both discriminative and domain-invariant, reliably addressing the issue of distribution shift in new data (with few or no labels) caused by factors such as changes in transformer operating conditions. Lastly, by comparing $Z_{3}$ and $Z_{4}$, it is evident that fine-tuning the model further enhances the precision of partial discharge recognition in the target domain.

To validate the superiority of the model proposed in this paper compared to classical models, different classical networks were selected and trained using source domain data. The accuracy of the source and target domain, compared to the proposed model, is shown in Table 3. To further validate the effectiveness and scalability of the proposed method, besides AlexNet, this paper also uses a KAN to improve the LeNet and ResNet models combined with a DANN. The results of source and target domain recognition accuracy are also shown in Table 3.

From Table 3, we can see that the method combining AlexNet-KAN with a DANN achieves a recognition accuracy in the target domain that is higher than that of all the classical models presented in the table. Furthermore, after improving LeNet and ResNet using the proposed method, their recognition accuracy in the target domain has also been enhanced, indicating that the proposed method has a certain degree of generalizability.

In engineering practice, the electromagnetic environment at substations is complex, and pulse-type interference, similar to discharge pulses, can easily be incorrectly included in the PRPD spectrum. To simulate the real operating conditions of transformers, salt-and-pepper noise is added to the target domain PRPD feature fingerprint maps.

Based on different signal-to-noise ratios (SNR), salt-and-pepper noise is added to the target domain. The transformer partial discharge recognition accuracy for $Z_{1}$ and $Z_{4}$ on the target domain is shown in Table 4.

As shown in the table above, under the same noise interference conditions, $Z_{4}$ significantly outperforms $Z_{1}$ in terms of recognition accuracy on the target domain, further validating the generalization capability of the recognition model proposed in this paper.

## 4. Conclusions

To address the issues of distribution shift and the lack of or scarcity of labels in newly collected transformer partial discharge data, which result in insufficient performance of existing recognition models, this paper proposes a transformer partial discharge domain adaptation recognition method that combines AlexNet-KAN with a DANN. This paper analyzes the effectiveness and scalability of the proposed method and adds salt-and-pepper noise to the target domain to verify the model’s applicability in practical engineering scenarios. The following conclusions are drawn:

1. Using a KAN to improve AlexNet can enhance the expressive capability of the transformer partial discharge recognition model, thereby increasing the accuracy of the model in identifying transformer partial discharge data;
2. To enhance the model’s recognition accuracy for new, unlabeled transformer partial discharge data with distribution shifts, the domain adversarial mechanism is introduced into AlexNet-KAN, resulting in a domain-adaptive transformer partial discharge recognition model combining AlexNet-KAN with a DANN. This model employs the domain adversarial mechanism to extract features with both discriminative and domain-invariant properties from both the source and target domains, thereby better aligning the data distributions between the source and target domains. It reliably addresses the issue of low recognition accuracy in the original partial discharge recognition model due to distribution shifts and the presence of unlabeled or sparsely labeled new data, effectively integrating deep learning and DDANs in the field of transformer partial discharge recognition;
3. To further validate the effectiveness and scalability of the method, a comparison is made between the transformer partial discharge recognition model that combines AlexNet-KAN with a DANN (the proposed method) and classical models, as well as classical models improved by the proposed method. This comparison is conducted on data with no distribution shift and on data with distribution shift and missing labels. The results show that the proposed method outperforms classical methods in terms of partial discharge recognition rates and demonstrates a certain degree of scalability;
4. After adding noise interference to the target domain, the proposed method maintains a high level of partial discharge recognition accuracy on the target domain, further validating the effectiveness of the method presented in this paper.

## Figures and Tables

**Table 1:** Network parameter setting.

	Layer Type	Number of Layers	Channels	Convolution Kernel (Stride)
AlexNet	Convolutional Layer + Batch Normalization Layer + Max Pooling Layer	5	64	3 × 3 (2)
	Fully Connected Layer	3	128, 64, 4	
AlexNet-KAN	Convolutional Layer + Batch Normalization Layer + Max Pooling Layer	5	64	3 × 3 (2)
	KAN Layer	3	128, 10, 4	

**Table 2:** Comparison of four scenarios.

No.	Source Domain Recognition Accuracy	Target Domain Recognition Accuracy
$Z_{1}$	95.32%	42.46%
$Z_{2}$	98.75%	45.73%
$Z_{3}$	98.14%	78.48%
$Z_{4}$	97.62%	86.85%

**Table 3:** Comparison with classical models and comparison with classical models improved by the proposed method.

Model Name	Source Domain Recognition Accuracy	Target Domain Recognition Accuracy
LeNet	92.96%	35.82%
The combination of LeNet-KAN and DANN	94.84%	66.28%
ResNet	94.95%	38.71%
The combination of ResNet-KAN and DANN	96.28%	79.26%
AlexNet	95.32%	42.46%
The combination of AlexNet-KAN and DANN	97.62%	86.85%

**Table 4:** Comparison of recognition rates under different signal-to-noise ratios.

SNR/dB	Target Domain Recognition Accuracy	
	$Z_{1}$	$Z_{4}$
20	40.57%	84.76%
18	36.12%	82.35%
16	27.46%	76.64%

## Data Availability

The original contributions presented in the study are included in the article, further inquiries can be directed to the corresponding author.
